# La drépanocytose en Guyane : bilan de 30 années de dépistage néonatal (1992-2021)

**DOI:** 10.48327/mtsi.v4i1.2024.488

**Published:** 2024-02-13

**Authors:** Narcisse ELENGA, Vathanaksambath RO, Joddy MAFEMA MISSINDU, Noelis THOMAS BOIZAN, Tania VAZ, Aude LUCARELLI, Marie Élise ARMOUDON-FLERET, Solange BUENDÉ

**Affiliations:** 1 Service de médecine et chirurgie pédiatrique, Centre hospitalier de Cayenne, Cayenne, Guyane; 2 Centre intégré de drépanocytose, Centre hospitalier de Cayenne, Cayenne, Guyane; 3 Association Drépaguyane, Cayenne, Guyane; 4 Service de néonatologie, Centre hospitalier de l'Ouest guyanais Franck Joly, Saint-Laurent-du-Maroni, Guyane

**Keywords:** Syndromes drépanocytaires majeurs, Dépistage néonatal, Incidence, Guyane, Amérique du sud, Sickle cell disease, Neonatal screening, Incidence, French Guiana, South America

## Abstract

**Introduction:**

La drépanocytose est l'une des maladies génétiques les plus fréquentes en France. En Guyane, le dépistage néonatal a été mis en place en 1992, en même temps que les autres programmes de dépistage des maladies infantiles. L'objectif de cette étude est de décrire l'organisation et les résultats du dépistage de la drépanocytose en Guyane entre 1992 et 2021.

**Matériels et méthodes:**

Nous avons utilisé plusieurs sources de données : les données issues du Programme de médicalisation des systèmes d'information (PMSI), recueillies depuis 2005, les rapports d'activité du Programme national de dépistage néonatal et les données des campagnes de dépistage organisées par l'association Drépaguyane entre 2010 et 2021 sur 1 300 sujets. Les échantillons de sang des nouveau-nés sont collectés par prélèvement capillaire ou veineux et absorption sur papier buvard (Guthrie) en même temps que ceux des autres dépistages néonatals. Les papiers séchés sont envoyés au laboratoire interrégional de Lille pour être analysés. À Saint-Laurent-du-Maroni, afin de réduire la proportion de perdus de vue, un double dépistage est réalisé et le résultat est rendu avant la sortie de maternité. Les données recueillies anonymement ont été analysées à l'aide du logiciel STATA.

**Résultats:**

Parmi les 175 593 naissances entre 1992 et 2021, le dépistage a permis de détecter 823 syndromes drépanocytaires majeurs et 17 950 hétérozygotes. Les syndromes drépanocytaires majeurs comprennent 493 de formes homozygotes SS (60 %), 302 SC (37 %) et 28 S-Bêta-thalassémie (3 %). L'incidence des syndromes drépanocytaires majeurs dans la population des nouveau-nés est de 1/213, IC 95 % [1/236-1/204] et celle des hétérozygotes de 1/10, IC 95 % [1/12-1/8]. La majorité de ces enfants (52 %) était originaire de l'Ouest guyanais. Le délai entre le dépistage et les résultats des tests était de 7 jours. Seuls les résultats pathologiques (homozygote, hétérozygote) sont communiqués aux parents et/ou au médecin traitant par courrier. Ces données confirment la tendance à l'augmentation du nombre d'enfants dépistés pour syndromes drépanocytaires majeurs en Guyane. Les données issues des campagnes de dépistage organisées par l'association Drépaguyane ont permis de décrire la répartition des différentes fractions d'hémoglobines anormales et de confirmer que l'HbS est plus fréquente dans l'ouest de la Guyane.

**Conclusion:**

La Guyane est le territoire français où l'incidence des syndromes drépanocytaires majeurs est la plus élevée, et cette incidence continue d'augmenter au fil du temps. Ces données seront utilisées pour guider les politiques de santé publique dans la poursuite de l'amélioration des soins et de la prévention primaire.

## Abstract (en)

**Background:**

Sickle cell disease is one of the most common genetic diseases in France. In French Guiana, neonatal screening was introduced in 1992, at the same time as other screening programs for childhood diseases. The aim of this study is to describe the organization of newborn screening for sickle cell disease in French Guiana.

**Materials and methods:**

We used several data sources: data collected from hospital records since 2005, activity reports from the national neonatal screening program and data from screening campaigns organized by the Drepaguyane association between 2010 and 2021 on 1,300 subjects. Blood samples from newborns are collected by capillary or venous sampling and absorbed on blotting paper (Guthrie) at the same time as those for other neonatal screenings. The dried papers are sent to the inter-regional laboratory in Lille, for further processing. In Saint-Laurent-du-Maroni, in order to reduce the proportion of people lost to follow-up, a double screening is carried out and the results are returned before discharge from the maternity hospital. All data were entered into an anonymous Excel file. The data were analyzed using STATA software.

**Results:**

Among the 175,593 screened neonates between 1992 and 2021, screening detected 823 infants with sickle cell disease and 17,950 heterozygotes. Sickle cell genotypes include 493 SS (60%), 302 SC (37%) and 28 S-Beta-thalassemia (3%). The incidence of sickle cell disease was 1/213, 95% CI [1/236-1/204], and that of heterozygotes 1/10, IC 95% [1/12-1/8]. The majority of these children (52%) were from the Maroni region. The delay between screening and test results was 7 days. Only pathological results (homozygous, heterozygous) were communicated to parents and/or the attending physician by post. These data confirm the upward trend in the number of children screened for sickle cell disease in French Guiana. Data from screening campaigns organized by the Drepaguyane association have enabled to describe the distribution of the various abnormal hemoglobin fractions, and to confirm that HbS is more frequent in Western French Guiana. In Cayenne, in 2021, the active file comprised 699 patients, including 266 children under 18 years old.

**Discussion and conclusion:**

This study provides valuable data on 30 years of neonatal screening for sickle cell disease in French Guiana, and on the evolution of sickle cell disease patients. It confirms that French Guiana is the French territory with the highest incidence of sickle cell disease. This incidence continues to rise over time. The study reveals the improvement in the organization of sickle cell disease management in French Guiana between 1992, when screening was introduced, and the present day. It highlights the role of patient associations in the fight against this disease, by organizing awareness and screening campaigns. These data will be used to guide public health policies in the pursuit of improved care and primary prevention.

## Introduction

La drépanocytose est la maladie génétique la plus fréquente dans le monde. C'est une maladie héréditaire de l'hémoglobine caractérisée par la présence d'une hémoglobine anormale : l'hémoglobine S (S pour l'anglais *sickle* = faucille). Celle-ci est due à la mutation d'un gène localisé sur le chromosome 11 codant pour l'un des deux types de protéines qui forment l'hémoglobine : la bêta-globine (Tableau I) [18]. Les syndromes drépanocytaires majeurs (SDM) regroupent la drépanocytose homozygote SS, la plus fréquemment rencontrée, et les hétérozygoties composites associant l'HbS à une autre anomalie de l'hémoglobine - HbS/β-thalassémie, HbS/HbC, HbS/HbD-Punjab ou HbS/HbO-Arab, S-Lepore, AS Antilles (forme dominante) [22]. Il s'agit d'une maladie grave, dont la morbidité est considérable. En l'absence de prise en charge efficace, 50 à 80 % des enfants décèdent avant l’âge de 5 ans, dans les pays à forte prévalence des SDM et offrant peu de possibilités de prise en charge [5]. Le pronostic des enfants et adultes atteints de SDM s'est considérablement amélioré grâce au dépistage néonatal et la mise en place des mesures prophylactiques au stade pré-symptomatique de la maladie [20]. La drépanocytose est la maladie dépistée la plus fréquente dans la population française [4, 12, 14].

**Tableau I:** Formules moléculaires des hémoglobines humaines normales Molecular formulas of human normal hemoglobins

		Adulte	Nouveau né
HbA	α_2_ β_2_	97 %	15 - 30 %
HbA2	α_2_ δ_2_	2 - 3 %	Traces
HbF	α_2_ γ_2_	< 1 %	70 - 85 %

En Guyane, territoire français situé en Amérique du Sud, avec une population très diversifiée composée d'Amérindiens, d'Afrodescendants, d'Asiatiques et d'Européens, le dépistage néonatal généralisé de la drépanocytose a été mis en place en 1992. La Guyane est reconnue comme ayant la plus haute incidence des SDM de toute la France [4]. Jusqu’à ce jour, aucune étude ne s'est penchée sur l'organisation et les résultats de ce dépistage. Cette étude a pour objectif de décrire l'organisation du dépistage de la drépanocytose en Guyane entre 1992 et 2021.

Le diagnostic prénatal consiste à rechercher l'allèle muté dans l'ADN fœtal à partir de cellules du placenta dès la 12^e^ semaine de grossesse ou par amniocentèse vers la 16^e^ semaine. Il est également possible de réaliser un diagnostic préimplantatoire (DPI) sur des embryons obtenus par fécondation *in vitro,* mais ce procédé est lourd et très encadré juridiquement.

Le dépistage néonatal consiste à identifier parmi tous les nouveau-nés ceux qui sont susceptibles d’être atteints de maladies rares, graves et le plus souvent génétiques ainsi que la surdité [13, 20]. Aujourd'hui, le dépistage néonatal vise à détecter 13 maladies [8]. Bénéficiant d'un diagnostic précoce, ces nouveau-nés auront accès à un traitement pouvant modifier le cours de l’évolution de leur maladie avant que n'apparaissent des lésions irréversibles.

## Matériels et méthodes

### Lieu de l’étude

La Guyane est un territoire d'outre-mer français bordé au nord par l'océan Atlantique sur 320 km environ, à l'ouest par le Suriname (520 km de frontière commune), au sud et à l'est par le Brésil (580 km de frontière commune). Le fleuve Oyapock constitue la frontière est, et le Maroni la frontière avec le Suriname. En 2021, la population de la Guyane était estimée à 294 150 habitants (INSEE 2021). Cette population est très mobile entre Guyane, Suriname, Guyana et Brésil [1]. En raison de sa population afro-descendante, la Guyane est une région où l'incidence de la drépanocytose est élevée.

### Sources de données

En Guyane, le dépistage de la drépanocytose, commencé en 1992, concerne tous les nouveau-nés. Le test est effectué au Centre régional de dépistage néonatal Hauts-de-France, à l'hôpital Jeanne de Flandre au Centre hospitalier universitaire de Lille, en même temps que le dépistage des autres maladies infantiles. Dans cette étude qui s’étend de 1992 à 2021, nous avons utilisé plusieurs sources de données : a) une base de données extraite du PMSI des trois principaux hôpitaux de Guyane et des thèses d'exercice de médecine, b) les rapports d'activités du Programme national du dépistage néonatal et c) les données des campagnes de dépistage organisées par l'association de lutte contre la drépanocytose Drépaguyane entre 2010 et 2021 sur 1 300 sujets âgés de 18 à 70 ans, dont 60 % de femmes, résidant à Cayenne, Kourou, Matoury, Saint-Georges, Sinnamary, Iracoubo, Mana, Saint-Laurentdu-Maroni, Apatou, Grand-Santi, Maripasoula et Papaïchton. Le résultat de l’électrophorèse de l'hémoglobine a montré 10,5 % de formes AS, 0,4 % de formes SC, 0,2 % de formes SS et 0,2 % des formes AC et AE. La numération formule sanguine ne fait pas partie du bilan de dépistage de masse. Les personnes dépistées avec SDM sont orientées vers l'hôpital pour leur suivi.

**Figure 1:**
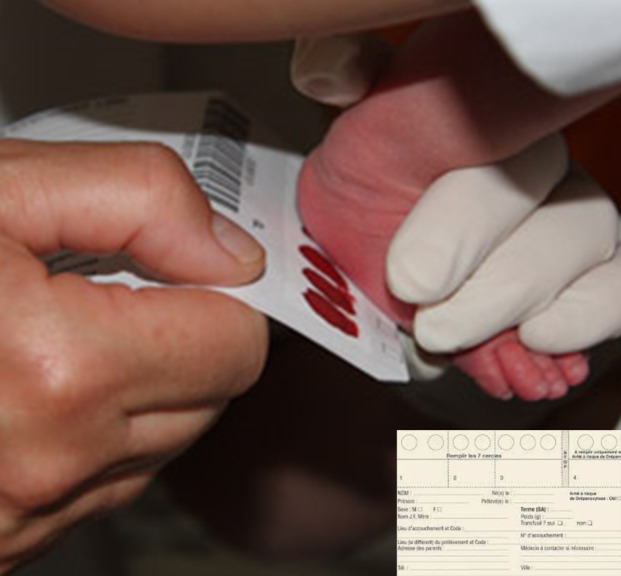
Prélèvement sur papier buvard pour le test de Guthrie Sampling on blotting paper for Guthrie test

Les parents doivent donner leur consentement oral à la réalisation des tests de dépistage et donc avant le prélèvement. Les échantillons de sang des nouveau-nés sont collectés par prélèvement capillaire (Fig. 1) ou veineux et absorption sur papier buvard (Guthrie) en même temps que ceux des autres dépistages néonatals. Les papiers séchés sont envoyés par voie postale au laboratoire interrégional de Lille.

### Les différents intervenants de ce dépistage

Ce dépistage généralisé est réalisé au sein de chaque maternité :

- les maternités de Guyane : Centre hospitalier de Cayenne, cliniques Véronique et Saint Paul à Cayenne (les maternités de Cayenne assurent la moitié des naissances), Centre médico-chirurgical de Kourou (1/6 des naissances), Centre hospitalier de l'Ouest guyanais (1/3 des naissances);
- les Centres délocalisés de prévention et de soins (CDPS) dans les communes isolées, gérés par les centres hospitaliers du littoral, pour les rares naissances dans ces centres.

Les sages-femmes, puéricultrices et infirmières de maternité s'occupent du prélèvement. Les papiers buvards sur lesquels sont déposées les gouttes de sang prélevées aux talons des nouveau-nés au troisième jour de vie sont directement acheminés au laboratoire dédié au dépistage néonatal.

### Déroulement du dépistage réalisé en Guyane

Toutes les naissances vivantes sont concernées. Les prélèvements sont envoyés par voie postale, par chaque maternité, au laboratoire interrégional de Lille. Les résultats sont transmis aux maternités de naissance ainsi qu'au centre régional de dépistage. Le centre régional de dépistage recueille les résultats et les transmet directement par courrier aux familles et aux médecins traitants. Ces médecins sont amenés à rencontrer, informer et conseiller les familles, à assurer la prise en charge de l'enfant. Une puéricultrice de la protection maternelle et infantile (PMI) assure le suivi des patients à domicile et la recherche des perdus de vue.

Les CDPS ont également la responsabilité de l'information et du suivi des patients, en collaboration avec les services de pédiatrie des Centres hospitaliers de Cayenne ou de l'Ouest guyanais. Le service de néonatologie du Centre hospitalier de Cayenne et le service de pédiatrie du Centre hospitalier de l'Ouest guyanais assurent le dépistage des nouveaunés hospitalisés.

Les médecins de ville constituent un relais essentiel entre le centre de PMI, la famille du nouveau-né, le centre de référence de la drépanocytose pour Cayenne et le service de pédiatrie du Centre hospitalier de l'Ouest guyanais. À Saint-Laurent-du-Maroni, un médecin généraliste de ville assure le suivi de quelques enfants drépanocytaires.

### Méthodes biologiques utilisées

**Figure 2:**
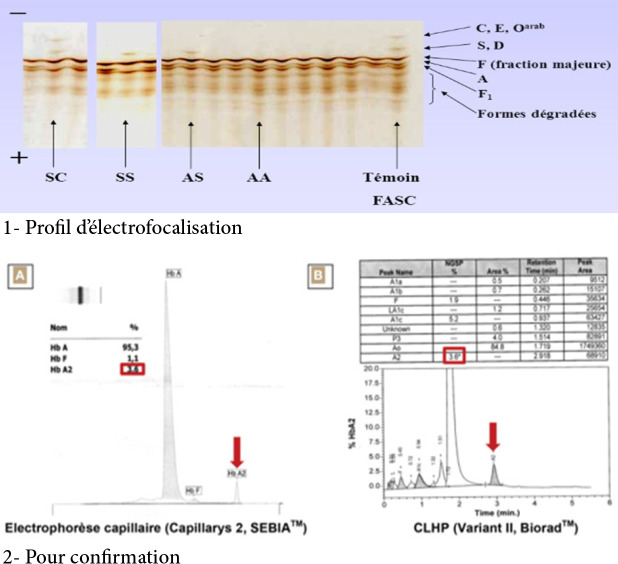
Techniques d’électrophorèse de l'hémoglobine Hemoglobin electrophoresis techniques

Historiquement, l'isoélectrofocalisation (IEF) de l'hémoglobine dans un gradient de pH 6 à 8, qui permet de mettre en évidence une différence de point isoélectrique entre la protéine mutée et la protéine normale, a été largement utilisée [15] (Fig. 2). Cette technique permet d'observer une différence de migration entre l'Hb S et l'Hb S Antilles, l'Hb D ou l'Hb G. Elle permet de dépister la présence d'Hb S même à des taux très bas (moins de 5 %), ce que ne permet pas la méthode électrophorétique standard sur acétate de cellulose à pH alcalin. Actuellement, la technique utilisée est la spectrométrie de masse type MALDI TOF suivie du contrôle par chromatographie liquide haute performance (HPLC). Ces techniques automatisées permettent, d'une part, de séparer les hémoglobines en fonction de leur point isoélectrique, et d'autre part de les quantifier.

### Double dépistage à Saint-Laurentdu-Maroni

Afin de réduire la proportion de perdus de vue, un double dépistage est réalisé dont le résultat est rendu avant la sortie de maternité. Ce double dépistage qui ne concerne que les nouveau-nés de mères hétérozygotes ou présentant un SDM, est réalisé sur sang total du nouveau-né ou sang du cordon. La technique utilisée est l'HPLC (Test d'Itano). La confirmation génétique se fait à Pointe-à-Pitre, soit 20 tests par an.

### Dépistage dans les Centres délocalisés de prévention et de soin

Très peu d'accouchements ont lieu dans ces CDPS. Comme à l'hôpital, le prélèvement se fait au 3^e^ jour sur papier buvard. Ces buvards sont regroupés et envoyés par voie postale à Lille. Le résultat est retourné par mail au centre de PMI et au CDPS de naissance.

### Méthodes statistiques

L'ensemble des données a été introduit sur un fichier Excel totalement anonyme. Ces données ont été analysées à l'aide du logiciel STATA (Stata Statistical Software : Release 15. College Station, TX : StataCorp LP), permettant de calculer les moyennes de chaque type d'hémoglobine par localité de naissance.

## Résultats

Parmi les 175 593 nouveau-nés qui sont nés de 1992 à 2021, le dépistage a permis de détecter 823 SDM et 17 950 hétérozygotes (Tableaux I et II). Aucun refus parental n'a été constaté. Les SDM comprennent 493 formes homozygotes SS (60 %), 302 SC (37 %) et 28 S-Bétathalassémies (3 %). À noter 34 cas d'hémoglobinose C (l'hémoglobinose C n'est pas un SDM). L'incidence des SDM sur population totale est de 1/213, IC 95 % [1/236-1/204] et celle des hétérozygotes de 1/10, IC 95 % [1/12-1/8] (Tableau III). Nous n'avons pas noté a de cas de forme AS Antilles.

**Tableau II:** Résultats du dépistage de la drépanocytose en Guyane (1992-2021) Results of newborn screening for sickle cell disease in French Guiana (1992-2021)

Formes des SD		Formes majeures			Formes mineures					
Années	Nombre Total	Hb SS	Hb SC	Hb Sbthal,	Hb CC	Hb AS	Hb AC	Hb AD	Hb AE	Autres
1992	2 562	9	5	0	1	168	59	0	0	16
1993	3 871	6	3	0	1	233	98	8	4	3
1994	4 012	7	7	0	0	245	75	8	6	2
1995	3 937	9	2	1	2	271	73	0	2	3
1996	4 282	3	2	0	2	275	100	9	4	3
1997	4 244	13	3	1	0	315	90	12	5	1
1998	4 591	13	6	1	1	361	102	7	1	4
1999	4 672	12	11	0	0	367	104	7	1	4
2000	5 124	14	7	0	1	372	117	16	0	1
2001	5 374	14	8	3	1	388	130	13	2	6
2002	5 382	19	13	1	0	385	118	9	0	8
2003	5 470	17	10	2	1	399	127	4	3	32
2004	5 583	12	7	0	0	440	113	8	5	15
2005	5 940	23	9	0	0	477	144	1	2	0
2006	6 184	14	14	1	4	515	167	0	9	2
2007	6 315	22	6	0	1	470	147	0	0	0
2008	6 124	11	11	2	3	525	159	0	0	0
2009	6 167	11	13	0	2	449	149	0	0	0
2010	6 176	14	8	1	3	486	159	0	0	0
2011	6 220	12	11	0	3	491	173	0	0	0
2012	6 599	15	12	0	0	531	161	0	0	0
2013	6 463	20	13	1	4	545	199	0	0	0
2014	6 654	18	11	3	0	551	181	0	0	0
2015	6 795	16	12	0	0	561	173	0	0	0
2016	7 193	22	15	2	0	587	222	0	0	0
2017	7 400	28	15	1	4	722	238	0	0	0
2018	8 042	26	19	0	0	698	222	0	0	0
2019	8 068	31	20	3	0	700	-	-	-	-
2020	8 004	33	16	5	0	708	-	-	-	-
2021	8 149	29	13	0	0	770				
**Totaux**	**175 597**	**493**	**302**	**28**	**34**	**14 005**	**3 080**	**102**	**44**	**100**

Nous n'avons pas noté de forme AS Antilles Hb Sbthal : Hb S/β-thalassémie. SD : syndrome drépanocytaire

**Tableau III:** Incidence des syndromes drépanocytaires majeurs en Guyane (d'après les données du Tableau II) Incidence of major sickle cell syndromes in French Guiana (based on data in Table II)

Phénotype	% IC 95 %
Hb SS	0,28 [0,23-0,30]
Hb SC	0,17 [0,14-0,19]
HbSpThal	0,018 [0,007-0,021]

Ces enfants appartiennent majoritairement à des populations originaires du Maroni (Tableau IV). Le délai entre le dépistage et le résultat est de 7 jours. Seuls les résultats pathologiques (homozygotes, hétérozygotes) sont communiqués à la maternité de naissance, au centre de référence (CID) ou de compétences (Saint-Laurent-du-Maroni), aux parents et/ou au médecin traitant par courrier.

**Tableau IV:** Proportion des syndromes drépanocytaires majeurs par commune Proportion of major sickle cell syndromes by location

Années/SDM	Total	St Laurent	Cayenne	Kourou	St Georges	Papaïchton
1992	15	7	7	1	0	0
1993	10	3	6	1	0	0
1994	14	4	10	0	0	0
1995	14	4	8	2	0	0
1996	7	2	4	1	0	0
1997	17	4	12	1	0	0
1998	21	9	9	3	0	0
1999	23	15	6	2	0	0
2000	22	14	6	2	0	0
2001	26	10	14	2	0	0
2002	33	12	16	5	0	0
2003	31	15	13	2	1	0
2004	19	10	7	2	0	0
2005	32	21	8	3	0	0
2006	33	16	12	5	0	0
2007	29	14	14	1	0	0
2008	27	11	12	4	0	0
2009	26	15	7	4	0	0
2010	26	12	10	3	0	1
2011	20	14	6	0	0	0
2012	27	16	9	2	0	0
2013	38	17	18	3	0	0
2014	32	16	14	2	0	0
2015	30	15	12	3	0	0
2016	39	17	18	4	0	0
2017	43	14	17	8	0	4
**SDM/Nombre naissances (%)**	**654/143 334 (0,46)**	**307/53 734 (0,57)**	**275/73 100 (0,38)**	**66/14333 (0,46)**	**1/1 081 (0,93)**	**5/1086 (0,46)**

**Figure 3:**
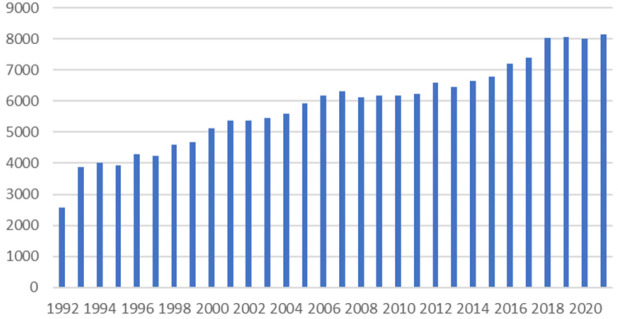
Évolution annuelle des grossesses en Guyane Annual evolution of pregnancies in French Guiana

Les Figures 3 et 4 confirment révolution croissante du nombre de grossesses et d'enfants dépistés avec un SDM en Guyane.

**Figure 4:**
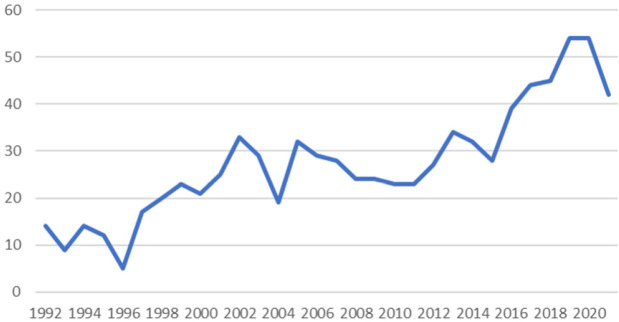
Évolution annuelle du nombre de naissances avec syndromes drépanocytaires majeurs en Guyane Annual trend in number of births with major sickle syndromes in French Guiana

**Figure 5:**
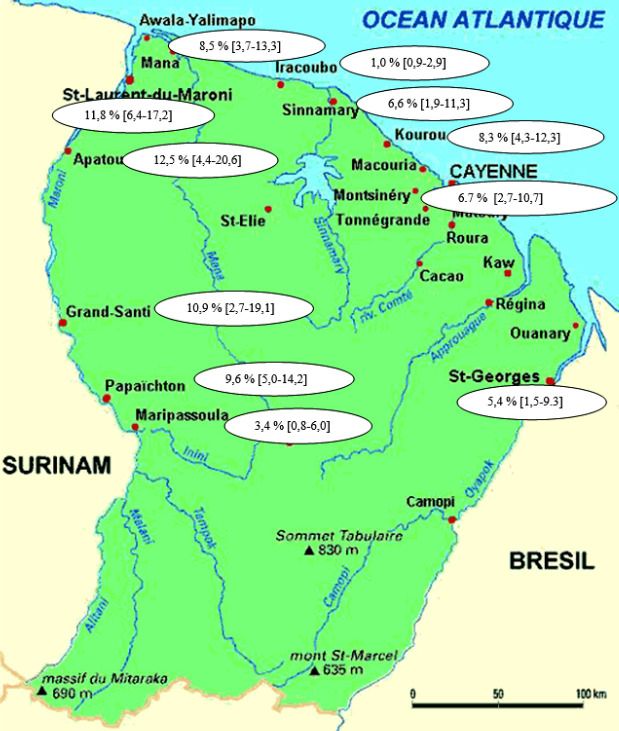
Répartition de l'allèle BS en Guyane (% et intervalle de confiance à 95 %) à partir du dépistage anonyme et volontaire de 1 300 sujets Distribution of the BS allele in French Guiana (% and 95% confidence interval) from anonymous and voluntary screening of 1,300 individuals

La Figure 5 est issue des campagnes de dépistage organisées par l'association Drépaguyane. Elles décrivent la répartition des différentes fractions des hémoglobines, avec l'Hb S plus présente dans l'ouest de la Guyane.

En 2021, la file active du CID (Centre intégré de drépanocytose) était composée de 699 patients dont 266 enfants de moins de 18 ans. Cinquante-deux patients vivaient à Maripasoula ou Papaïchton. Quarante-trois patients ont été vus pour la première fois en 2021. Dix-sept patients sont suivis pour hémopathie non maligne non drépanocytaire (thalassémies essentiellement). Quatre-vingt-huit patients sont traités par hydroxycarbamide dont 38 enfants et 50 adultes. Par ailleurs, le nombre de patients bénéficiant d’échanges transfusionnels itératifs est passé de 9 en 2014 à 20 en 2021. Une dizaine de patients proviennent de Kourou. Dix autres habitent Saint-Laurent-du-Maroni. Depuis l'ouverture du CID, plus de 300 nouveaux patients ont été pris en charge. En 2021, le dépistage néonatal a permis la découverte de 15 nouveaux cas (9 SS, 4 SC, 2 Sβ+).

## Discussion

En 30 ans de dépistage néonatal sur près de 200 000 nouveau-nés, cette étude a permis de déterminer l'incidence des SDM en Guyane. Elle confirme ce qui était attendu : la Guyane est le territoire français où l'incidence des SDM est la plus élevée, loin devant les Antilles et Mayotte [4]. Elle est proche de celle de certains pays d'Afrique équatoriale [17, 19]. Cette incidence élevée peut s'expliquer par les origines africaine et indienne des populations ayant peuplé la Guyane. Comme le montrent le Tableau IV et la Figure 5, la répartition géographique des hémoglobinopathies ne semble pas homogène en Guyane française. L'hémoglobine S est prédominante dans les populations bushinengués, descendants des marrons noirs, c'est-à-dire des esclaves qui ont pour la plupart fui les plantations de la Guyane hollandaise voisine aux xviii^e^ et xix^e^ siècles pour se réfugier dans la forêt. Ces populations ont créé des sociétés autonomes en marge du monde colonial, à l'intérieur du Suriname et le long du fleuve Maroni en Guyane française. Ces sociétés sont présentées comme héritières de la culture de leurs ancêtres ouest-africains ou centrafricains, y compris l'endogamie [16]. La ville de Kourou compte également une forte proportion de Bushinengés. Dans l'agglomération de Cayenne, ce sont les personnes d'origine haïtienne qui sont les plus touchées par l'hémoglobine S. En revanche, dans la population amérindienne de l'est de la Guyane, l'hémoglobine S est moins fréquente, la thalassémie étant l'hémoglobinopathie la plus fréquente. Une étude réalisée en Guyane a montré que les haplotypes prédominants étaient béninois et bantous [23]. Ce dépistage a permis d'introduire des mesures prophylactiques précoces chez les nouveaunés et les nourrissons atteints, et d’éduquer les parents à reconnaître les symptômes des complications cliniques, afin de recourir à une consultation médicale d'urgence si nécessaire. La plupart des nouveau-nés dépistés font l'objet d'un suivi. Le double dépistage à Saint-Laurent-du-Maroni permet de poser un diagnostic avant la sortie de maternité, mais de nombreuses mères vivent au Suriname et ne reviennent pas pour le suivi de leur enfant atteint de SDM. Ces enfants étaient souvent perdus de vue. Ils n’étaient revus (aux urgences de Saint-Laurent-du-Maroni) qu’à l'occasion de complications de la maladie. Depuis 2011, l'IDE coordinatrice de l'ETP organise, avec l'aide d'un médiateur en santé, un entretien avec ces femmes avant leur sortie de maternité. Elle tient également un fichier des coordonnées des parents, et est en mesure de convoquer la majorité des enfants dans le circuit de soins. Par ailleurs, l'amélioration continue de la coopération sanitaire entre la Guyane et le Suriname a également permis un meilleur suivi des femmes enceintes surinamaises venant accoucher à Saint-Laurentdu-Maroni.

La méthode de dépistage sur papier buvard est celle utilisée dans toute la France, et les techniques utilisées sont celles recommandées par la Haute autorité de santé (HAS) [7]. Cependant, ces techniques ont leurs limites : certaines situations sont à l'origine de faux négatifs, comme les grands prématurés, pour lesquels le dépistage est effectué trop tôt. À ce stade, les mutants de l'hémoglobine sont exprimés à des niveaux très faibles, à la limite de détection des techniques utilisées. Un autre problème, plus fréquent, est la transfusion de concentrés de globules rouges *in utero.* Cette transfusion peut fausser le résultat en montrant la présence d'une hémoglobine adulte normale, en fait d'origine transfusionnelle. Les faux positifs concernent des variants rares (Hope, HbK Woolwich, etc.) associés au variant S, donnant ainsi faussement lieu à un profil FS. Dans ces cas, le test d'hémoglobine doit être répété à distance [7]. Le dépistage néonatal de la drépanocytose a également permis d'identifier des nouveaunés hétérozygotes. Ces enfants hétérozygotes sont des porteurs sains et n'ont donc pas de bénéfice direct à être dépistés, mais leur dépistage a permis la mise en place d'un conseil génétique pour les familles concernées. En Guyane, le dépistage a toujours été généralisé, contrairement à la France hexagonale où ce dépistage était ciblé. Mais aujourd'hui, l'HAS recommande un dépistage généralisé pour tous les nouveau-nés [6]. L'incidence de cette maladie s'est accrue au fil du temps, entraînant une augmentation du nombre de cas, comme le confirment les données du dépistage au niveau national [3]. La drépanocytose représente aujourd'hui le premier risque génétique en France [9].

Concernant les hétérozygotes dépistés à la naissance, l'information est inscrite dans leur carnet de santé. Cette information se perd au fil des années, à tel point qu’à l'adolescence, on ne s'en souvient plus, et une fois devenu adulte, on s’étonne parfois d'avoir un enfant malade, sans avoir fait la moindre démarche pour réitérer l’électrophorèse de l'hémoglobine. L’électrophorèse de l'hémoglobine est systématiquement demandée pendant la grossesse. Lorsqu'une femme est hétérozygote, il est conseillé à son partenaire de subir une électrophorèse de l'hémoglobine pour déterminer son statut drépanocytaire, mais il refuse souvent ou ne se présente pas au dépistage.

Les campagnes de dépistage anonyme et volontaire menées par l'association Drépaguyane ont permis aux personnes nées avant 1992, année de mise en place du dépistage néonatal en Guyane, et à celles nées dans d'autres pays de la Caraïbe où le dépistage néonatal universel n'a pas encore été mis en place [12], de connaître leur statut drépanocytaire et de prendre des mesures de prévention primaire. Lors de la création du Centre intégré de drépanocytose (CID) en 2014, nous avons opté pour une collaboration directe avec les associations de malades. L'association Drépaguyane joue un rôle majeur dans la lutte contre la drépanocytose en Guyane. Sa mission principale est de sensibiliser et d'accompagner les malades, avec pour objectif global d'informer massivement le grand public, afin de favoriser le dépistage et d'améliorer le quotidien des malades. Avec son programme de sensibilisation dans les écoles, l'association aide les enseignants et les élèves à mieux comprendre cette maladie, et donc à mieux accueillir les enfants drépanocytaires. Les membres de Drépaguyane travaillent aussi en étroite collaboration avec les équipes médicales et paramédicales du CID pour améliorer le suivi des patients et optimiser l'observance thérapeutique. Les visites à domicile, l'aide aux formalités administratives et le soutien communautaire sont autant d'activités qui permettent à de nombreux patients de rester dans le circuit de soins. Enfin, Drépaguyane est aussi « la voix des patients » auprès des équipes soignantes et de la direction des hôpitaux de Guyane. Les associations de patients atteints de drépanocytose se sont fortement mobilisées pour que cette maladie soit reconnue comme une priorité mondiale [17].

Pour les enfants atteints de SDM, l'organisation des soins en Guyane permet que le suivi soit bien organisé et structuré dans les trois grands hôpitaux de Guyane. Concernant les enfants vivant dans les communes isolées, le suivi est organisé par les médecins travaillant dans les CDPS. Ces enfants sont hospitalisés en pédiatrie pour leur bilan annuel. Cette approche se heurte cependant au problème des patients migrants qui, à leur arrivée en Guyane, subissent des contraintes sociales liées à la maladie et à leur parcours. Ces patients représentent la moitié des patients pris en charge. Avec l'accroissement de la précarité en Guyane depuis l’épidémie de Covid-19, notamment au sein de ces populations migrantes, celles-ci ont de plus en plus de mal à rester dans le système de soins [2]. Pourtant, après avoir intégré ce dernier, elles peuvent bénéficier d'un suivi gratuit et d'une intensification thérapeutique qu'elles n'ont pas dans leur pays d'origine. La Guyane étant un territoire à forte immigration, le problème doit être abordé de manière globale [10, 11]. Les points forts de cette étude résident dans l'exhaustivité du dépistage sur 30 ans, avec des résultats consistants. L'organisation des soins, qui suit les mêmes standards qu'en France métropolitaine, permet de proposer aux patients dépistés pour un SDM une prise en charge conforme aux recommandations internationales.

## Conclusion

Cette étude fournit des données sur 30 ans de dépistage néonatal de la drépanocytose en Guyane et sur l’évolution de la prise en charge des personnes atteintes de SDM vivant en Guyane. Elle confirme que la Guyane est le territoire français où l'incidence des SDM est la plus élevée et que cette incidence continue d'augmenter au fil du temps. L’étude montre également l'amélioration de l'organisation de la prise en charge de la drépanocytose en Guyane entre 1992, date de la mise en place du dépistage, et aujourd'hui. Elle met en évidence le rôle des associations de patients dans la lutte contre cette maladie, en organisant des campagnes de sensibilisation et de dépistage. Ces données seront utilisées pour guider les politiques de santé publique dans la poursuite de l'amélioration des soins et de la prévention primaire.

## Contribution des auteurs

EN et RV : conception de l’étude, rédaction et correction du manuscrit.

MJ, TN, VT, LA et BS : révision et validation du protocole, recueil des données, analyse, rédaction et correction du manuscrit.

AME : recueil des données, rédaction et correction du manuscrit.

## Conflits d'intérêts

Les auteurs n'ont aucun conflit d'intérêts à déclarer.
